# Development and Psychometric Evaluation of the Gender Identity Scale for Transgender Women in China

**DOI:** 10.3389/fpsyg.2021.792776

**Published:** 2022-01-04

**Authors:** Meng Han, Bailin Pan, Yuanyuan Wang, Amanda Wilson, Runsen Chen, Rengang Wu

**Affiliations:** ^1^Department of Medical Psychology, The School of Health Humanities, Peking University, Beijing, China; ^2^Department of Plastic Surgery, Transgender Clinic, Peking University Third Hospital, Beijing, China; ^3^Division of Psychology, Faculty of Health and Life Sciences, De Montfort University, Leicester United Kingdom; ^4^Vanke School of Public Health, Tsinghua University, Beijing, China

**Keywords:** transgender women, gender identity, scale development, psychometrics, factor analysis, Chinese

## Abstract

Transgender women are an important subgroup of the transgender umbrella and have their own unique gender identity. This article aimed to understand and measure the latent concept of gender identity among Chinese transgender women from a multi-dimensional perspective. Through a two-phase, iterative scale development process, we developed the Gender Identity Scale for Transgender Women (GIS-TW) in Chinese. Literature reviews, expert consultations, and focus groups constitute phrase 1 of the study, which resulted in the first version of GIS-TW with 30 items. In phrase 2, exploratory factor analysis on a sample of 244 Chinese transgender women revealed a six-factor solution across the 22 items. The Bem Sex Role Inventory was included to test for convergent validity, and the Rosenberg Self-Esteem Scale was used to test discriminant validity. Then we conducted the confirmatory factor analysis with an independent sample of 420 Chinese transgender women, which produced the final version of GIS-TW with 21 items. The internal consistency (Cronbach’s alpha = 0.71–0.87) and test-retest stability (*r* = 0.73–0.87) of each factor was good. In conclusion, the GIS-TW is a reliable and valid psychometric tool for the assessment of Chinese transgender women’s gender identity. Future application of the scale will help transgender women obtain better gender confirmative interventions.

## Introduction

Transgender is an umbrella term typically used to describe individuals whose gender identity is different from their sex assigned at birth and/or their gender identity is outside the limits of the sex binary (Kozee et al., 2012). The terminology “transgender women” refers to those individuals whose gender identity is women but who were assigned as male at birth. In the assessment of gender identity, transgender women and transgender men are usually subsumed under the umbrella of the term transgender, with little or no focus on the subtle differences between them. However, during the process of medically transitioning and social role transitioning, there are many differences between transgender women and transgender men, not only due to the direction of their transition, but also within their neuroanatomy (Mueller et al., 2021), fertility preservation (Amir et al., 2020), social acceptance (Jaffee et al., 2016), and sexual experiences (Crespi et al., 2008).

Numerous innovations in research with transgender individuals have highlighted the multiple dimensions of gender identity development and the diversity that exists among transgender individuals (Levitt and Ippolito, 2014; Forcier et al., 2020). Scholars suggest that a multi-dimensional measurement of gender identity should be a framework when researching transgender communities to capture the subtle differences of a person’s gender identity (Dickey et al., 2016). Consistent with Johnson’s research (2012), a transgender identity formation should be regarded as an ongoing process when transitioning (Johnson, 2012), and this transition should be continuously evaluated. The need for more nuanced measurements of gender identity provides a rational for the development of Gender Identity Scale for Transgender Women (GIS-TW). The researchers therefore drew from a broad range of theoretical frameworks, previous research, and clinical experiences to develop and test the GIS-TW Chinese version of the scale. In this current study, we aimed to understand and measure the latent concept of gender identity among transgender women from a multi-dimensional perspective. A multi-dimensional perspective allowed for scale items to be developed that understand the support required for a needs assessment by transgender health services who are providing gender-affirming medical interventions, including hormone treatment and surgery, as well as providing clinicians with a measurement to screen for the distress that may be experienced by transgender women (Reisner et al., 2015).

Trans-Identity theory further provided a foundational perspective during the development of the scale (Nagoshi et al., 2014). According to Trans-Identity theory, an individual’s gender identity is considered as a continually dynamic interaction among three factors. The first is an embodied aspect of the self that generates changes in bodily experiences. The second part is an explicitly self-constructed aspect of gender identity, which derives meaning from the narrative of one’s life experiences during transition. In Trans-Identity theory, the third factor influencing the formation of gender identity is the socially constructed aspect of identity, which has external factors that restrict gender identity. Specifically, social environment factors relating to sex role restricts individuals and can act as a restricting factor as the individual may feel pressure to comply with the expectations of a certain category of gender, such as men are breadwinners. These restricting factors of the social environment act on and are influenced by the formation of a person’s gender identity. When the individual behaviors and performances are restricted in conformity with these expectations, it promotes an abductive social construction of gender identity that is constantly being negotiated and renegotiated both between the individual and their social world (Nagoshi and Brzuzy, 2010). Trans-Identity theory provides a theoretical basis to generate new items.

Sexual experiences can also play an important role in the formation and development of a person’s gender identity. In addition, the evaluation of an individual’s sexual attraction can also be an important part of their body image (Grogan, 2016). The perplexity of body image and sexual attraction can also highlight emotional problems, especially the worry about a sexually related body part, such as relating to an individual being self-consciousness over the body part and self-actualizing whether a different individual is sexually attractive to them during a sexual act (Dharma et al., 2019). Part of the complexity includes feeling sexually attractive in itself is a positive feeling (Liss et al., 2011), which can in opposition increase the level of self-esteem, at least in the short term (Breines et al., 2008). However, in these studies, the evaluation of individual sexual attractiveness is carried out under the framework of a duality of birth-assigned males and females only, and the attraction by cis men is taken as an indicator to measure the attractiveness of cis women, which can lead to the sexualization of women (Calogero and Siegel, 2019). Therefore, it is believed that when evaluating the impact of sexual attraction among transgender women the researchers should consider transgender women’s unique subjective feelings, like self-esteem and sexual attraction, and focus on how this promotes or inhibits the construction of one’s transgender identity.

Pleasure and satisfaction as part of sexual behavior and sexual attraction are also the important aspects of sexual health for transgender women (Reis et al., 2021). Sexual pleasure refers to the ability to take a positive attitude toward one’s own sexual behavior and the ability to experience happiness during sexual behaviors (Bond et al., 2020). It is a basic component of sexual health and well-being (Ford et al., 2019). The study of cisgender women found that the subjective experience of sexual behavior was related to a higher level of well-being (Zimmer-Gembeck and French, 2016). Women dissatisfied with their body during sexual intercourse reported that they were less self-confident, avoided sexual behaviors more frequently, and showed a lower sense of self-efficacy when having an orgasm (Wiederman, 2000; Yamamiya et al., 2006). The majority of research on transgender sexual health does not pay attention to the sexual pleasure of transgender people, let alone transgender women, and this is an oversight by researchers given the central importance of pleasure to understanding sexual behaviors and family planning behaviors (Bradford and Spencer, 2020). Depending on where the transgender person is in their transition and what transitions they want to undergo there may be a lack of confidence required to enjoy sexual pleasure (Cerwenka et al., 2014). For example, if their identity affirmation includes vaginoplasty they may not have the confidence to enjoy sexual pleasure until they have undergone the surgery (De Cuypere et al., 2005). Therefore, gender dysphoria related to their genitals could result in emotional distance from the physical pleasure associated with orgasm, potentially leading to feelings of disconnectedness or disgust associated with orgasm or the inability to orgasm (Chadwick et al., 2019; Bradford and Spencer, 2020). Sexual pleasure is important to promote sexual well-being and improve people’s quality of life. Therefore, this study considers the sexual pleasure of transgender women as a dimension of the evaluation.

The current study reports the development and psychometric evaluation of the GIS-TW scale within the Chinese transgender women population. It is necessary to consider the gender identity of transgender women within the theoretical framework of the above three factors, so that their experiences can be fully understood and meaningfully integrated into the scale adaption and validation. The scale was developed to measure the different aspects of gender identity for transgender women in China before, during, and after transition in order to improve gender-affirming medical treatment. Such a tool is essential to provide transgender health service professionals a screening tool to conduct targeted and comprehensive assessment so transgender women can qualify for gender-affirming care and any distress can be reduced.

## Materials and Methods

### Participants

This study used a cross-sectional design. Participants were recruited using two methods. First, some of the participants were directly recruited in-person from the transgender clinic of the Third Hospital of Peking University, Beijing, China. The recruitment poster was also distributed within the participants’ networks to help share the study with other transgender women who did not use the clinic, their friends, or other community members. Therefore some of the participants were recruited from the transgender community in the form of snowball sampling. The inclusion criteria included (a) Self-identified as a transgender women, (b) age 18 and above, (c) and currently living in China. The data were collected in two stages; Sample 1 was collected from May to December 2019, while sample 2 and sample 3 were collected from March to December 2020.

Sample 1 was used for single item analysis and Exploratory Factor Analysis (EFA). A total of 257 answers were collected for this sample. However, from this sample, 13 answers were excluded from analysis because participants gave an incorrect response to the attention check. The content of the item was a direct instruction to the participants: “Please directly select the third option for this question.” If the participants selected an option other than the third option, it meant that they gave an incorrect response to the attention check and were not included leaving 244 transgender women in the sample. The final sample ranged in age from 18 to 43 years with a mean of 22.18 (SD = 5.60). Sample 2 was used for Confirmatory Factor Analysis (CFA). A total of 453 answers were collected in this sample. With sample 2, 23 answers were excluded from analysis because participants incorrectly responded to the attention check (same as sample 1). The final analytic sample included 420 individuals, ranging in age from 18 to 39 years with a mean of 21.18 (SD = 4.10). Sample 3 was used for temporal stability. 80 participants, who were also included in the analysis in sample 2 were selected and re-measured at an interval of 1 month. All of the 80 individuals’ answers were included in the analysis. In order to verify the internal consistency and reliability of the scale, we combined sample 1 and sample 2 for analysis.

### Measures

#### Scale Development

**Figure 1:**
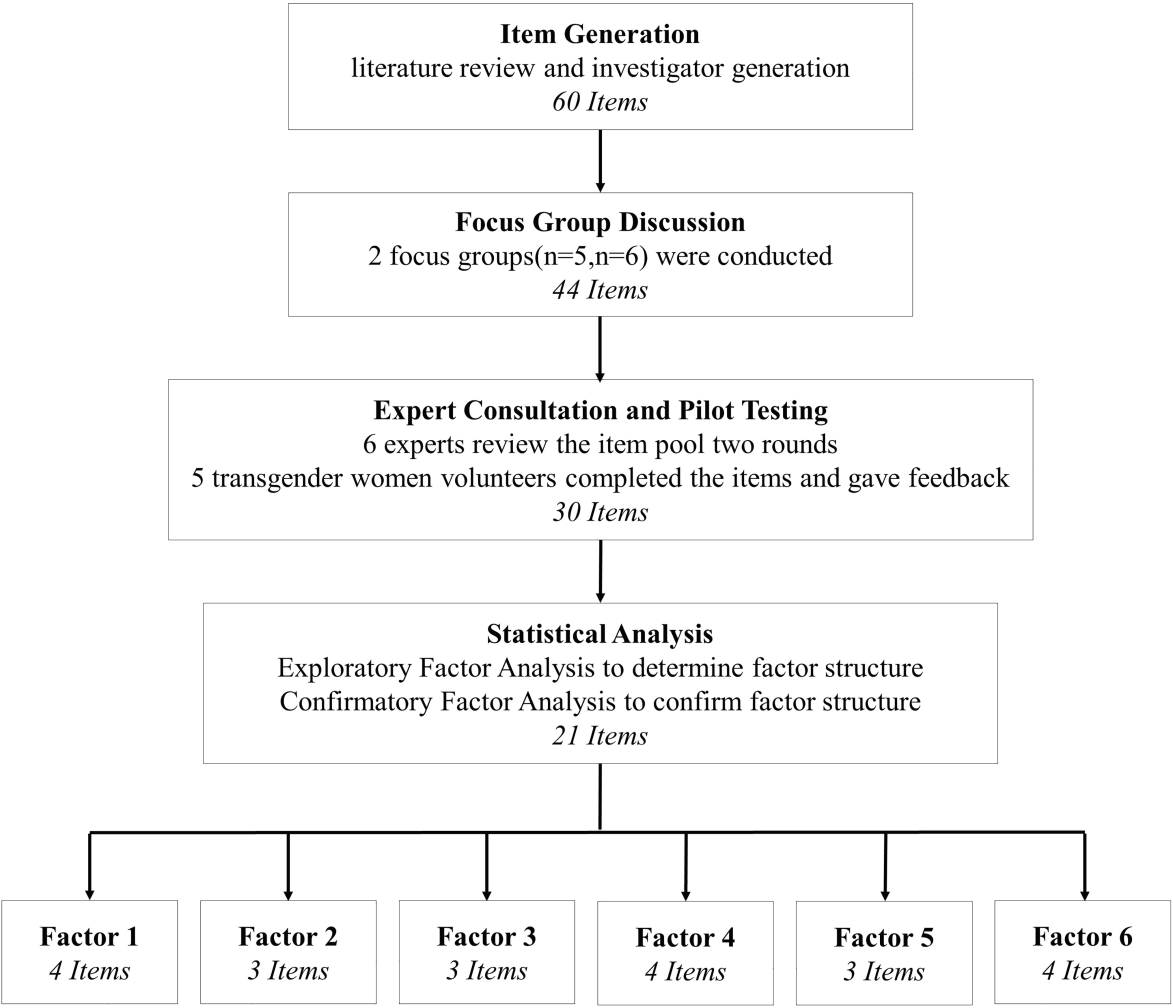
Flowchart of the Gender Identity Scale for Transgender Women (GIS-TW) development.

A two-phase, iterative scale development process was used to generate the GIS-TW Chinese version (Figure 1). The first phase of the study was devoted to the development of the initial scale, which included the three steps of item generation, expert review, and pilot testing. The second phase aims to verify the reliability and validity of the newly developed scale by testing it within independent samples using statistical analysis.

As a first step in the scale development process, a thorough review of the related literature and extant questionnaires on gender identity was conducted. Based on the literature reviewed, we determined a multi-dimensional model of defining gender identity was appropriate, consisting of dimensions falling under the three broad conceptual categories identified above. Secondly, during the process of constructing the item pool, the first author (Chinese cisgender male) translated the items of the existing scales into Chinese, then the Chinese version items were back translated by a Master’s student majoring in English (Chinese cisgender female). To address any problems that could arise due to translation, the first author revised the translation and invited another Master’s student majoring in English (Chinese cisgender female) to do the back translation again. This step ensured that the Chinese translation for these items was accurate and easy to understand. Using questions from existing scales and studies, along with novel items to reflect the unique sexual experience of transgender women and the various dimensions of the target construct, the first author generated a pool of 60 items. Of the questions incorporated from existing scales, some were taken directly in addition to the scale, while others were modified from extant transgender identity scales (Cohen-Kettenis and van Goozen, 1997; Deogracias et al., 2007; Schneider et al., 2016). After the item pool was generated, the researchers conducted two focus groups to evaluate the surface validity of the items. The two focus groups included one group of five and another group of six transgender women who were recruited from the existing community links the researchers have with the LGBT center in Beijing. During the focus groups, participants reviewed and discussed each item to determine its appropriateness, evaluate the language translation to Chinese, and affirm its relevance within the conceptual framework of gender identity. At the suggestion of the focus group participants, we made several language changes to make the items more consistent with and sensitive to their experiences of transitioning. Since some of the items were adapted from a Western questionnaire, they did not conform to the characteristics of Chinese culture and were therefore replaced. In addition, additional scale items were removed based on a unanimous consensus of irrelevance from the transgender women in the focus groups (Figure 1).

After revising the item pool from the focus group, the researchers invited six experts who worked within transgender communities for many years to give expert advice on the content validity of items. The experts included two psychiatrists, one endocrinologist, one plastic surgeon, one Professor of Clinical Psychology, and one expert in scale development. Each expert was asked to evaluate items for consistency, relevancy, and applicability to tailor the measure in light of their area of expertise, with a focus placed on assessing each item for brevity, clarity, and accuracy. The primary researcher revised item wording based on written and verbal feedback from each of the six experts. After the first round of expert consultation, the primary researcher conducted a close examination of their feedback and made revisions of the item pool accordingly. To check that the primary researcher understood the feedback correctly, the revised scale was sent to the experts for a final review. After the second round of experts’ evaluation and discussion the revised item pool finally formed the initial scale, which included 30 items. A Likert scale was chosen as the best item response form for the instrument and each item included five response options from “I strongly disagree” to “I strongly agree.”

A pilot test was conducted to test whether there were undetected problems in the initial scale. Five transgender women volunteers were recruited by the existing community who participated in testing the electronic version of the questionnaire, providing feedback on the length of questionnaire and time to complete, word usage, and clarity of the scale items. Therefore, the first phase of the scale development process resulted in the first version of the GIS-TW consisting of 30 items.

#### Bem Sex Role Inventory

The Bem Sex Role Inventory (Bem, 1974) was used to assess respondents’ endorsement of gender-stereotypical personality traits. It consists of 20 masculine gender role items and 20 feminine gender role items. Participants were asked to rate the degree to which these stereotypically masculine and feminine traits described their personality on a 7-point Likert scale, ranging from 1 (never or almost never true) to 7 (always or almost always true). The BSRI yields a masculinity score and a femininity score for each participant, with higher scores indicating either more masculine or feminine personality traits. This instrument has been validated and widely used with Chinese samples (Du et al., 2021; Lu et al., 2021). Both the Masculinity subscale (*α* = 0.83) and Femininity subscale (*α* = 0.80) showed good internal consistency in this study. The BSRI was used to test for convergent validity of the GIS-TW Chinese version.

#### Rosenberg Self-Esteem Scale

The Rosenberg Self-Esteem Scale is a widely used measure of global self-esteem developed by Rosenberg (1989). It is a 10-item scale which is rated on a four-point Likert scale, ranging from 1 (strongly disagree) to 4 (strongly agree). After reverse coding negatively worded items, a higher score indicates higher levels of self-esteem. The Chinese version of the scale has demonstrated evidence of reliability and validity in multiple studies (Li et al., 2020; Wang et al., 2021). In the current study, Cronbach’s alpha value of RSES was 0.85, which showed good internal consistency. The BSRI was used to test for discriminant validity.

### Demographics

Five questions were included pertaining to demographics: age, education, marital status (unmarried, married, divorced, and widowed), gender of preferred partner (male, female, both, none, others). In addition, we asked participants the age at which they first began to experience gender dysphoria.

### Procedures

The three scales (in the order of GIS-TW, BSRI, and RSES) and the demographic variables mentioned above, along with informed consent, formed the electronic questionnaire. The study was set up in such a way that clicking on the “next” button at the end of the informed consent would indicate participants’ agreement of consent and subsequently directed participants to the survey page. This study was approved by the ethics committee of Peking University Health Science Center. The item order of GIS-TW was randomized using a random integer set generator, and all participants were presented the items in the same randomized order. Based on findings from previous researcher (Meade and Craig, 2012), three attention check items were included as part of the questionnaire in order to ensure the quality and assurance of the data set. For the purpose of this study, participants were provided with the following definition of transgender: “a transgender women is defined as a person whose biological sex does not match their identity as female.” The survey took an average of 12 min to complete.

## Results

**Figure 2:**
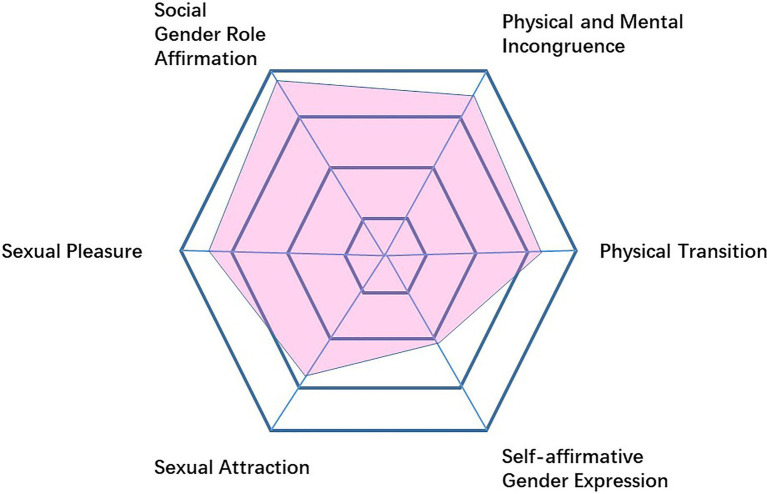
Participants score on six dimensions of GIS-TW.

A total of 710 individuals completed the online survey, and as mentioned above, in phase 1, 13 responses were excluded and 23 responses in phase 2 were excluded. Demographics of the three samples can be found in Table 1. The majority of the participants in the three samples were Han nationality and their relationship status was single. The most commonly reported education level was a Bachelor’s degree in all three groups, followed by a high school diploma. 64% of the participants in sample 1 reported that they had Gender Dysphoria before the age of 12, compared with 77.6% in sample 2. About 60% of the participants were currently receiving hormone replacement therapy. Participant scores in the whole sample highlighted that there were differences in scores between the six dimensions (see Figure 2). After data collection of sample 1, item-total correlation was first evaluated, then, item 3, item 19 and item 20 were removed because the correlation between these items and the total score was not significant as they were attention checks, leaving 27 items to undergo EFA.

**Table 1:** Demographic characteristics.

	Sample 1 (*N = 244*)		Sample 2 (*N = 420*)		Sample 3 (*N = 80*)	
	*n*	%	*n*	%	*n*	%
Age(mean ± SD, range)	22.18 ± 5.60, 18–43		21.18 ± 4.10, 18–39		21.44 ± 4.20, 18–38	
Nationality						
Han	238	97.5	405	96.4	79	98.7
Others	6	2.5	15	3.6	1	1.3
Education						
Less than high school	25	10.2	29	6.9	7	8.8
High school	55	22.5	96	22.9	17	21.3
Junior college	39	16.0	72	17.1	16	20.0
Bachelor’s degree	102	41.8	189	45.0	31	38.8
Advanced degree	23	9.4	34	8.1	9	11.3
Marital status						
Single	227	93.0	407	96.9	78	97.5
Married	12	5.0	11	2.6	2	2.5
Divorced	5	2.0	2	0.5	0	0
Widowed	0	0	0	0	0	0
The earliest age of Gender Dysphoria						
Before 6	57	23.4	143	34.0	25	31.3
6–12	99	40.6	183	43.6	36	45.0
13–18	68	27.9	83	19.8	11	13.8
After 18	20	8.2	11	2.6	8	10.0
Currently on HRT						
No	76	31.1	171	40.7	33	41.3
Yes	168	68.9	249	59.3	47	58.7
Gender of preferred partner						
Male	80	32.8	124	29.5	23	28.8
Female	72	29.5	147	35.0	25	31.2
Both	67	27.5	115	27.4	19	23.8
Others	25	10.2	34	8.1	13	16.2

### Exploratory Factor Analysis

After completing data screening, we used the data of sample 1 to complete EFA on the remaining 27 items. The principal axis factor method is used to extract 7 factors with eigenvalues greater than 1.0, and the optimal oblique rotation (Promax with Kaiser Normalization) method is used to rotate the covariance matrix. According to the loading value of each item on each factor, the poorly performing items were deleted according to the following criteria: (1) loading value of each factor is less than 0.3; and (2) loading value greater than 0.4 on two or more factors. According to the principles of deleting one item at a time, 5 items were deleted and 22 effective items were retained, which were divided into 6 factors (see Table 2). The final EFA results showed that KMO-test value was 0.836, Bartlett spherical test result was significant (approximate chi-square = 1980.25, df = 231, *p* < 0.001) and the remaining items accounted for 63.44% of the total variance. Each of the 22 items had moderate to high factor loadings, ranging from 0.42 to 0.88. The factors are labeled according to the items content: Factor 1 labeled “Social Gender Role Affirmation” contained 4 items. Factor 2 labeled “Physical and Mental Incongruence” contained 3 items. Factor 3 labeled “Physical Transition” contained 4 items. Factor 4 labeled “Self-affirmative Gender Expression” contained 4 items. Factor 5 labeled “Sexual Attraction” contained 3 items. Factor 6 labeled “Sexual Pleasure” contained 4 items. Cronbach’s alpha for the overall scale was 0.89, indicating the reliability of this version of the scale was good.

**Table 2:** Exploratory factor analysis of the gender identity scale for transgender women.

GIS-TW Items	Factor loading					
	1	2	3	4	5	6
Factor 1: Social Gender Role Affirmation						
13. I want to participate in all kinds of daily activities as a transgender woman, such as entertainment, shopping, dining and so on.	**0.81**	0.08	−0.14	0.06	−0.11	0.04
12. I want to carry out my work activities as a transgender woman, such as going to work, meeting with colleagues and leaders, meeting clients and so on.	**0.80**	−0.00	−0.04	0.02	0.05	−0.12
7. I want to live my life as a transgender woman all the time.	**0.68**	0.13	0.10	−0.06	−0.02	−0.03
10. I wish that I could become pregnant.	**0.42**	−0.16	0.09	−0.07	0.11	0.20
Factor 2: Physical and Mental Incongruence						
1. I fantasize about myself as a transgender woman daily.	−0.03	**0.78**	−0.02	−0.06	0.11	0.11
9. I am a transgender woman living in a man’s body.	0.08	**0.76**	0.05	0.03	−0.05	−0.02
4. My body makes me feel that I am not a real woman	0.12	**0.66**	0.18	0.08	−0.06	−0.03
Factor 3: Physical Transition						
8. I want to remove my male reproductive organs, such as testicles and penis.	−0.08	0.04	**0.80**	−0.09	0.01	0.10
2. I am sure I’m moving in the direction of transsexuality.	−0.03	0.17	**0.72**	0.10	0.00	−0.03
5. I want to change my physical characteristics by taking estrogen.	0.04	−0.14	**0.63**	0.10	0.05	−0.17
26. I want to change my male secondary sex characteristics, such as Adam’s apple, beard, body hair and so on.	−0.04	0.07	**0.62**	−0.09	−0.02	0.09
Factor 4: Self-affirmative Gender Expression						
14. I have taken public transport as a transgender woman, such as taxi, train, plane and so on.	−0.05	−0.09	−0.07	**0.88**	0.06	0.10
15. I have checked in a hotel as a transgender woman.	−0.01	0.10	−0.10	**0.78**	0.05	0.01
17. I have appeared in different social situations as a transgender woman.	0.02	−0.01	0.10	**0.70**	−0.08	−0.04
18. Relatives or friends have seen my transgendered identity.	0.13	0.03	0.08	**0.56**	0.11	−0.02
Factor 5: Sexual Attraction						
30. When I appear feminine, I think I’m attractive.	−0.05	−0.07	0.02	0.01	**0.88**	−0.02
27. When I appear feminine, I think someone would want to have sexual contact with me, such as kissing or caressing.	−0.02	0.05	0.02	0.07	**0.80**	−0.09
28. When I appear feminine, I think I am sexually attractive to others.	0.05	0.05	−0.02	0.04	**0.76**	0.10
Factor 6: Sexual Pleasure						
22. I prefer the ways of having sex that makes me feel like a woman.	0.00	0.00	−0.00	0.05	−0.07	**0.78**
21. I wish I could have vaginoplasty to have sex with my partner.	0.12	−0.14	0.11	0.06	−0.07	**0.66**
23. When I masturbate by stimulating my genitals, I feel more comfortable if I imagine my body parts are womanly.	−0.24	0.23	−0.12	0.02	0.01	**0.64**
24. I wish I could have breast implants and be fondled by my partner, which makes me sexually excited.	0.20	0.07	−0.05	−0.11	0.13	**0.54**
Deleted Items						
3. I do not like dressing as a man.						
19. Wearing feminine clothes could improve my emotional state.						
20. Wearing beautiful women’s underwear would bring me sexual excitement.						
6. Putting on beautiful makeup will make me feel happy.						
11. It is easier for me to express my true feelings as a woman.						
16. When I speak, I would deliberately carry a female-specific tone and voice						
25. In my sexual dreams, I appear as a woman						
29. When I appear feminine, I think someone wants to dance with me.						

*N = 244. The extraction method was principal axis factoring with an oblique (Promax with Kaiser Normalization) rotation. Factor loadings above 0.30 are in bold*.

### Confirmatory Factor Analysis

Based on findings from the EFA, the six-dimensional Gender Identity Scale was confirmed through CFA using the data from sample 2. Since the data screening showed that the data of sample 2 did not conform to the multivariate normality, the maximum likelihood with robust standard errors (so-called Satorra-Bentler estimator) was used to conduct the CFA. In the measurement model, error measurements were presumed not to be correlated and no indicator using double-loadings were permitted. The six factors were permitted to correlate based on evidence of factor interrelatedness from the original EFA. Appropriate cutoff values were assessed according to Hu and Bentler’s (1999) recommendations for good and adequate fit. Based on Hu and Bentler’s (1999) recommendations, criteria for an acceptable model fit is the comparative fit index (CFI) ≥ 0.90, Root Mean Square Error Approximation (RMSEA) ≤ 0.10, and Standardized Root Mean Square Residual (SRMR) ≤ 0.10 and criteria for good model fit is CFI ≥ 0.95, RMSEA≤0.06 and SRMR≤0.08 (Hu and Bentler, 1998, 1999). Modification indices and standardized residuals were then examined to identify localized areas of strain. Item 10 of Factor 1 was removed because of high modification index values. At this point, modification indices were examined to consider possible error covariance to attain greater parsimony. Two errors, Item 14 and Item 17, were permitted to correlate as there was reason to believe that there would be error covariance due to similar wording and close conceptual correspondence between the two items. Two additional errors, Item 3 and Item 23, were also permitted to correlate for the same reason.

The fitting index of the final model is between acceptable and good. The revised model (see Figure 1), specifying six factors (with 3 items loading on Factor 1, 3 items on Factor 2, 4 items on Factor 3, 4 items on Factor 4, 3 items on Factor 5, and 4 items on Factor 6). Six-factor covariance and two error covariance resulted in an interpretable model, sufficiently reproducing the observed relationship among indicators: *χ*^2^(df = 172, p<0.001) = 352.73, CFI = 0.93, RMSEA = 0.05 (90% CI 0.04–0.06), and SRMR = 0.06. Additionally, each of the 21 items had moderate to high factor loadings, ranging from 0.43 to 0.95, suggesting that the indicators were highly related to the purported factors (see Table 3).

**Table 3:** Confirmatory factor analysis of the GIS-TW.

GIS-TW Items	Factor loading						Cronbach’s alpha when item removed
	1	2	3	4	5	6	
Factor 1: Social Gender Role Affirmation							
13. I want to participate in all kinds of daily activities as a transgender woman, such as entertainment, shopping, dining and so on.	**0.80**						0.814
12. I want to carry out my work activities as a transgender woman, such as going to work, meeting with colleagues and leaders, meeting clients and so on.	**0.73**						0.818
7. I want to live my life as a transgender woman all the time.	**0.70**						0.818
Factor 2: Physical and Mental Incongruence							
1. I fantasize about myself as a transgender woman daily.		**0.66**					0.821
9. I am a transgender woman living in a man’s body.		**0.63**					0.823
4. My body makes me feel that I am not a real woman		**0.59**					0.820
Factor 3: Physical Transition							
8. I want to remove my male reproductive organs, such as testicles and penis.			**0.74**				0.818
2. I am sure I’m moving in the direction of transsexuality.			**0.66**				0.813
5. I want to change my physical characteristics by taking estrogen.			**0.60**				0.817
26. I want to change my male secondary sex characteristics, such as Adam’s apple, beard, body hair and so on.			**0.43**				0.825
Factor 4: Self-affirmative Gender Expression							
14. I have taken public transport as a transgender woman, such as taxi, train, plane and so on.				**0.76**			0.812
15. I have checked in a hotel as a transgender woman.				**0.69**			0.813
17. I have appeared in different social situations as a transgender woman.				**0.63**			0.810
18. Relatives or friends have seen my transgender identity.				**0.61**			0.815
Factor 5: Sexual Attraction							
30. When I appear feminine, I think I’m attractive.					**0.95**		0.815
27. When I appear feminine, I think someone would want to have sexual contact with me, such as kissing or caressing.					**0.90**		0.811
28. When I appear feminine, I think I am sexually attractive to others					**0.76**		0.814
Factor 6: Sexual Pleasure							
22. I prefer the ways of having sex that makes me feel like a woman.						**0.69**	0.821
21. I wish I could have vaginoplasty to have sex with my partner.						**0.69**	0.821
23. When I masturbate by stimulating my genitals, I feel more comfortable if I imagine my body parts are womanly.						**0.59**	0.821
24. I wish I could have a breast implants and be fondled by my partner, which makes me sexually excited.						**0.44**	0.824

*N = 420. Analysis: Estimator = MLM; χ^2^ (df = 172, p<0.001) = 352.734, CFI = 0.926, RMSEA = 0.05, SRMR = 0.06*.

### Reliability and Temporal Stability

In order to calculate the internal consistency of the scale more accurately, we combined the data of sample 1 and sample 2 in the final version of the scale. Cronbach’s alpha of each subscale included: Factor 1 was 0.73, Factor 2 was 0.76, Factor 3 was 0.71, Factor 4 was 0.80, Factor 5 was 0.87, Factor 6 was 0.75, and Cronbach’s alpha for the overall scale was 0.83, also demonstrating the reliability of the final version of the scale.

The test-retest reliabilities of the final GIS-TW Chinese version were assessed by using Sample 3. Randomly selected participants from sample 2 were asked to complete the final GIS-TW version again 1 month later (*n* = 80), and test-retest reliability was assessed by calculating the correlation coefficient between the scores of six subscales of two measurements. All six subscales were found to have good (> 0.7) temporal stability, as: Factor 1 was 0.84, Factor 2 was 0.73, Factor 3 was 0.87, Factor 4 was 0.86, Factor 5 was 0.75, and Factor 6 was 0.84.

### Construct Validity

In order to evaluate the convergent validity of the new scale, correlations (using Pearson’s coefficients) were examined between the GIS-TW scale and one of the previously validated scales (BSRI) measuring sex role. Because the contents measured by the six subscales were all related to transgender women, we expected that the correlation coefficients between the six subscales and the femininity in BSRI would be significant, but not with the masculinity. The results (see Table 4) showed that the six subscales were significantly correlated with femininity: a positive, small-to-medium correlation (as expected), thus demonstrating the scale’s convergent validity. But surprisingly, the subscale of Physical Transition showed a significant negative correlation with masculinity [r(244) = −0.20, *p* < 0.01]. Since the subscale measured the content of removing male secondary sex characteristics, it is reasonable that there is a small negative correlation between the subscale and a higher score on the masculinity subscale.

**Table 4:** Pearson’s correlation matrix between GIS-TW, BSRI, RSES.

Variable	*n*	*M*	*SD*	1	2	3	4	5	6	7	8	9
1. Social Gender Role Affirmation	244	17.20	2.91	1								
2. Physical and Mental Incongruence	244	13.26	2.01	0.38^**^	1							
3. Physical Transition	244	17.35	3.08	0.49^**^	0.31^**^	1						
4. Self-affirmative Gender Expression	244	10.74	4.78	0.27^**^	0.08	0.33^**^	1					
5. Sexual Attraction	244	9.73	3.98	0.27^**^	0.07	0.28^**^	0.54^**^	1				
6. Sexual Pleasure	244	17.18	2.81	0.38^**^	0.34^**^	0.21^**^	0.09	0.25^**^	1			
7. Masculinity	244	3.94	0.98	0.05	0.02	−0.20^**^	0.13	0.02	0.08	1		
8. Femininity	244	5.27	0.82	0.36^**^	0.27^**^	0.25^**^	0.37^**^	0.28^**^	0.24^**^	0.33^**^	1	
9. RSES	244	24.32	6.23	0.11	0.02	0.01	0.03	0.11	0.03	0.52^**^	0.34^**^	1

^*^ *p < 0.05;*

** *p < 0.01*.

Discriminant validity of GIS-TW was evaluated by examining the correlation coefficients between GIS-TW and one scale (RSES) assessing constructs that are theoretically unrelated to the gender identity of a transgender woman. It was expected that all six subscales would not correlate significantly with the RSES which measures global self-esteem. Pearson’s correlations showed that all six subscales were poorly correlated with the RSES [r(244) = 0.01–0.11, *p* = 0.08–0.97], thereby demonstrating discriminant validity of GIS-TW.

## Discussion

The identity of transgender women should not be measured simply through a single dimension, because gender identity includes both individual and social aspects, and of course, it has an inseparable relationship with sexual experience. No current scale measures the gender transition of transgender women using a multi-dimensional perspective. Therefore, we have developed the GIS-TW Chinese version and conducted a preliminary psychometric evaluation to ensure the scale measured the gender identity of transgender women from different aspects (i.e., before, during, and after transition), and the scale can be applied to clinical practice and screening for level of gender dysphoria, as well as for researchers. The generation of items is based on the extensive literature reviewed, the framework of the multi-disciplinary transgender identity, professionals’ cooperation, a combination of an additional Trans-Identity theoretical framework, and clinical practice, all derived from experts. Through a two-phase, iterative scale development process, the researchers verified the consistency between the item content and the theoretical hypothesis. Potential limitations in the scale were found, which were modified and retested in the follow-up process. Findings from the current study show that for Chinese transgender women, GIS-TW is a psychometrically sound, multi-dimensional instrument with demonstrated good reliability and validity. There were strong factor loadings and overall alpha coefficient values. Factor loadings were moderate to high on all indicators. The GIS-TW also evidences construct validity as demonstrated by its expected performance on tests of convergent and discriminant validity against theoretically related and unrelated constructs. Overall, suggesting that the construct of a transgender identity is not already captured by other measures of bias.

Gender Identity Scale for Transgender Women builds upon prior transgender identity scales and shows improvement when compared in three ways. First, the samples of this study were recruited in two ways, including participants from the transgender clinic and participants from the transgender woman’s community, which ensured the diversity of the sample. Because this scale is designed for measuring gender identity of all transgender women in China, the increase of sample diversity is conducive to the accuracy of the evaluation. Secondly, the scale can measure the six dimensions of transgender women using 21 items, making it the shortest scale that exists to date, and an efficient scale as it measures the most dimensions (6) when compared to the current gender identity scales. Shorter scales mean lower rejection rates, less attention loss, and less missing data (Harel et al., 2019). Finally, the advantage of GIS-TW over the previous scale is that it reflects the multi-dimensional construction of Chinese transgender women’s gender identity. This multi-dimensional construction of gender identity has been recommended for use by the literature (Bauer et al., 2017), but it is lacks incorporation within the current gender identity scales. The six dimensions contained in this scale are discussed below.

Social Gender Role Affirmation mainly focusing on the desire of transgender women to live as a transgender female in part of their social life, corresponds to the social construction of identity in the Trans-Identity Theory (Nagoshi et al., 2014). As noted above, there is a proven relationship between social gender identity and well-being (Zitelny et al., 2021). The researchers agree that transgender women can establish their own gender roles and gender expression in the way they like. However, in a society dominated by binary cisgender women, it may be an attempt to integrate into a social group. Although this may be partly a manifestation of internalization transphobia, it also has a certain degree of social adaptability, particularly in the case of sex role expectations.

The main concern of Physical and Mental Incongruence is the inconsistent feelings caused by the differences between one’s gender and having an assigned birth’s sexual anatomy. Both “gender dysphoria” of DSM-V and “gender incongruence” of ICD-11 take the inconsistent feelings experienced by transgender people as one of the key points of diagnosis and distress (Beek et al., 2016). As the suffering caused by inconsistency affects most transgender people, assessing the degree of incongruence is also an important aspect that can be addressed using the GIS-TW Chinese version. Physical Transition reflects the individual’s attitude toward removing their own male secondary sex characteristics and obtaining vaginoplasty or breast implants at the physiological level, which is also the core criteria of both the DSM-V and ICD-11 diagnosis (Beek et al., 2016). In addition, the physical transition through HRT or surgery is the main purpose behind the majority of transgender women’s decision to come into a clinic and contact with clinicians (Racila, 2021). The evaluation of this dimension could be used by clinicians who have transgender women clients to understand the individual’s attitude toward changing their physical characteristics through hormones and surgery.

Self-affirmative Gender Expression evaluated transgender women’s attempts to express their transgender identity, and it is also the only dimension to evaluate objective behavior of GIS-TW. According to the recommendation in the “Standards of Care for the Health of Transsexual, Transgender, and Gender Nonconforming People” published by The World Professional Association for Transgender Health (WPATH), “living part time or full time in another gender role, consistent with one’s gender identity” is one of the treatment options for gender dysphoria (World Professional Association for Transgender Health, 2012). That is, the expression of one’s transgender identity is a kind of confirmation of one’s gender identity at the behavioral level. Therefore, evaluating different situations where transgender identities can be expressed constitutes an important part of understanding the transgender women’s identity.

Sexual Attraction measures transgender women’s self-evaluation of their own sexual attractiveness as a transgender woman. Individual sexual attractiveness is an important part of their body image (Hockey et al., 2021), and it can also affect the level of an individual’s self-esteem (Kennis et al., 2021). The researchers believe that, for transgender women, subjective feelings of their own sexual attraction are more related to their transgender identity. When individuals express their transgender womanly characteristics, it is not only important for the social construction of their identity, but also for the recognition of their body image. Experiencing pleasurable sex is not only physically satisfying, but helps an individual realize some self-fantasies that result in arousal and identity development (Kiguwa, 2015). People may increase sexual arousal and sexual pleasure through role playing (Langdridge and Lawson, 2019). Therefore, Sexual Pleasure measures whether the self-affirming hormones and/or surgery can bring more sexual arousal and pleasure. Because the existing research rarely involves the experience of transgender people in sexual activities, it is necessary to further improve the exploration of sexual behavior and pleasure of different transgender subgroups in a sensitive way in the future research.

Through using GIS-TW a better understanding of the gender identity of transgender women can be formed using a comprehensive perspective. In addition, each sub-dimension can be used separately to measure different aspects of gender identity as the transgender women transition, as well as before and after transition. Participant scores in the current sample show that in general the scores of all dimensions are medium to high, and the dimension with the highest average score is Social Gender Role Affirmation, which reflects the sample group’s strong desire for living as a transgender woman in social life. The average score that was the lowest was in the dimension of Self-affirmative Gender Expression, meaning that the proportion of the sample group who actually tried to live their social life as a transgender women is low (see Figure 2). This may be due to the fact that most of the subjects in this study were individuals in the early stage of transitioning, of which 40% had yet to start hormone therapy, while 60% of the individuals who started hormone therapy on average had been doing so for a duration of 18.1 months, which is not long in the process of transitioning if the end goal is gender-affirming surgery. Therefore, future research needs to further explore the measurement performance of GIS-TW in transgender women during the later stages of transitioning, including transgender woman-based groups who have been using HRT long-term and transgender women groups who have received gender-affirming surgery. It is equally important to capture in the future the experience of those who do not want to undergo gender-affirming surgery or use hormones and have been living as a transgender woman for a long period of time.

While the GIS-TW demonstrates adequate reliability and validity, a number of limitations should be considered. While the study population was demographically diverse and the sample size is large, the scale was evaluated in a convenience sample, which lacks representativeness of the general Chinese transgender female population. Due to the same recruitment link for both samples, we cannot accurately calculate the proportion of participants recruited through the individual recruitment methods, which may also lead to a lack of representativeness. Additionally, the present study did not include information about several demographic characteristics that may have been useful, such as socioeconomic status, urban/rural distinctions, or whether the transgender identity has been disclosed to anyone or not. Further, while the results of convergent validity are acceptable, the correlation coefficients between the subscales and BSRI are low. This is because the content measured by each subscale is different, so using the BSRI to verify the convergent validity cannot reflect the contents measured by each subscale. In the future, when researchers apply different subscales of the GIS-TW Chinese version, they can test the convergent validity of the subscales by correlating content-related scales, which could also provide further support for the validity of this scale. Besides, future longitudinal research examining the predictive validity of the scale, such as whether different scores of subscales have different predictive effects on mental health, would also broaden the application of this scale. The scale also was developed and validated in the Chinese context, so it may be more suitable for Chinese transgender women. The English version needs further adaption and validation within Western samples. At last, we notice that the words of some items in the current version of the scale may show cisgenderism, such as “living in a man’s body” or “male reproductive organ.” In future iterations of the research, we will revisit the appropriateness of the wording of these items and make changes to minimize cisgenderism.

In conclusion, GIS-TW shows good psychometrics in the current study. It has multiple dimensions to understand the gender identity of transgender women when compared to previous questionnaires, and the measurement content is more detailed and comprehensive, making up for the lack of relevant content. With the increasing visibility of the transgender population and their expected demand for mental and physical care, GIS-TW will also be applicable to help medical professionals better understand their transgender women clients. It can provide guidance for clinical gender confirmative interventions, and improve the quality of transgender health services. Additionally, employing GIS-TW to explore possible correlates, such as age, education, earliest age of onset of gender dysphoria, and gender of preferred partner to different dimensions of gender identity is also warranted. In future studies, the scale can be used to explore possible variations of different stages of transition, such as before and after disclosure of transgender identity or before and after sexual reassignment surgery.

## Data Availability Statement

The raw data supporting the conclusions of this article will be made available by the authors, without undue reservation.

## Ethics Statement

The studies involving human participants were reviewed and approved by The Ethics Committee of Peking University Health Science Center. The patients/participants provided their written informed consent to participate in this study.

## Author Contributions

MH and RW designed the study. MH and BP were in charge of sample recruitment and data collection. MH carried out the statistical analysis, designed the figures, and wrote the first draft of the manuscript. YW, AW, and RC made substantial contributions to the revision of the manuscript. All authors have read and approved the final version of the manuscript.

## Conflict of Interest

The authors declare that the research was conducted in the absence of any commercial or financial relationships that could be construed as a potential conflict of interest.

## Publisher’s Note

All claims expressed in this article are solely those of the authors and do not necessarily represent those of their affiliated organizations, or those of the publisher, the editors and the reviewers. Any product that may be evaluated in this article, or claim that may be made by its manufacturer, is not guaranteed or endorsed by the publisher.
